# Electrostatic repulsive out-of-plane actuator using conductive substrate

**DOI:** 10.1038/srep35118

**Published:** 2016-10-07

**Authors:** Weimin Wang, Qiang Wang, Hao Ren, Wenying Ma, Chuankai Qiu, Zexiang Chen, Bin Fan

**Affiliations:** 1 State Key Laboratory of Optical Technologies on Nano-Fabrication and Micro-Engineering, Institute of Optics and Electronics, Chinese Academy of Sciences, Chengdu 610209, China; 2 School of Electrical, Computer, and Energy Engineering, Arizona State University, Tempe, Arizona 85281, USA; 3 College of Communication Engineering, Chengdu University of Information Technology, Chengdu 610225, China; 4 School of Optoelectronic Information, University of Electronic Science and Technology of China, Chengdu 610054, China

## Abstract

A pseudo-three-layer electrostatic repulsive out-of-plane actuator is proposed. It combines the advantages of two-layer and three-layer repulsive actuators, i.e., fabrication requirements and fill factor. A theoretical model for the proposed actuator is developed and solved through the numerical calculation of Schwarz-Christoffel mapping. Theoretical and simulated results show that the pseudo-three-layer actuator offers higher performance than the two-layer and three-layer actuators with regard to the two most important characteristics of actuators, namely, driving force and theoretical stroke. Given that the pseudo-three-layer actuator structure is compatible with both the parallel-plate actuators and these two types of repulsive actuators, a 19-element two-layer repulsive actuated deformable mirror is operated in pseudo-three-layer electrical connection mode. Theoretical and experimental results demonstrate that the pseudo-three-layer mode produces a larger displacement of 0–4.5 μm for a dc driving voltage of 0–100 V, when compared with that in two-layer mode.

Micro out-of-plane electrostatic actuators are among the most important microelectromechanical systems (MEMS) devices, and have been widely applied in radio frequency (RF) MEMS^1^,^2^,^3^,^4^ and optical MEMS^5^,^6^,^7^. Compared with thermal and piezoelectric actuators, electrostatic actuators have the advantages of fast response, low power consumption, and hysteresis-free characteristics^8^,^9^.

In general, electrostatic actuators have fixed and movable electrodes at different voltages. Most current out-of-plane electrostatic actuators, including parallel-plate and vertical comb-drive actuators, are based on the electrostatic attractive force that exists among electrodes with different voltages. The stroke of this type of actuator, i.e., maximum achievable displacement, is severely limited by the gaps between electrodes. This displacement is then determined entirely through fabrication processes. As a result, the out-of-plane surface-micromachined electrostatic actuators that were presented in prior studies have a micron-scale or even submicron-scale stroke because of the small gaps between the electrode layers. These layers are accumulated one by one on substrate surfaces with thicknesses that are limited by the nature of deposition processes. Numerous techniques have been developed to increase the stroke of out-of-plane surface-micromachined electrostatic actuators, including nonlinear flexures^10^,^11^, leveraged bending^12^,^13^, and pre-stress self-assembly^14^,^15^. However, these techniques are still based on the implementation of an attractive force. Moreover, their strokes cannot exceed the gap size, and the resulting actuators suffer from several problems, such as pull-in instability, stiction, and mechanical breakdown. These problems are associated with conventional attractive actuators when the strokes approach the gap size.

In contrast to these electrostatic attractive-force actuators, electrostatic repulsive-force actuators drive the movable electrodes away from the fixed electrodes. Their strokes can exceed the gap size, and these devices have been applied in translation and rotation micromirrors^16^,^17^. To the authors’ knowledge, the out-of-plane repulsive phenomenon in in-plane comb-drive actuators was first observed in 1990^18^,^19^, where it is called “levitation”. Figure 1a illustrates that the levitation phenomenon of the movable finger occurs because the potential of the substrate is the same as that of the movable finger. After a decade, Lee *et al*. introduced the term “repulsive-force actuators” for an in-plane repulsive-force actuator^20^. In 2003, He *et al*. proposed an out-of-plane repulsive-force actuator based on another electrode configuration^21^,^22^. Figure 1b,c show these two types of electrostatic repulsive actuators and it is easy to see that all three devices are based on asymmetric electric field distributions and realize similar functions. Firstly they have fixed electrodes and movable electrodes with electrical potential difference applied between them. In this paper, we name these electrodes the attracting electrode (AT) and actuated electrode (AC). Each side of AC is attracted by AT, and the attractive forces are denoted by *f*_*L*_, *f*_*R*_, *f*_*U*_, and *f*_*D*_ in Fig. 1. The direction and the size of the solid line arrows indicate the direction and magnitude of the attractive forces involved. Among these forces, *f*_*L*_ and *f*_*R*_ cancel each other out (Fig. 1a,c) or are counteracted by an external force (Fig. 1b). Thus, the movable electrode can only move in the directions of *f*_*U*_ or *f*_*D*_. Second, one side of AC is presented to a fixed electrode with the same potential, thereby changing the field distribution and suppressing the applied attractive force to the aforementioned side of AC, *f*_*D*_. This electrode is called the suppressing electrode (SU). Finally, the attractive force on the opposite side of AC, *f*_*U*_, moves AC away from SU, thereby offering the impression that AC is repulsed by SU. AC is not actually driven if only SU and AC are present. According to the perspective of the three electrode types, electrostatic attractive actuators only have ATs and ACs.

These repulsive actuators are suitable for surface micromachining processes and require two structural layers. Rezadad *et al*. recently proposed a three-layer repulsive actuator, as shown in Fig. 1d 23,24. In essence, the three-layer structure is space efficient in the lateral direction, but requires thick sizes in the vertical direction. It therefore allows high fill factors and is beneficial for specific applications.

Various electrostatic repulsive actuators have been presented. However, most of these actuators lack theoretical models, which are critical for guidance in design and optimization. In this study, a novel pseudo-three-layer repulsive actuator is proposed and analyzed through a physical model developed here. Based on this model, the effect of varying structural parameters on the electrostatic force of the actuator and the comparison of three repulsive actuators is summarized. At last the proposed actuator is validated with experimental measurements based on a 19-element deformable mirror (DM) that was developed through commercially available multi-user MEMS processes (MUMPs)^25^.

## Results

### Pseudo-three-layer repulsive actuator

**Figure 1:**
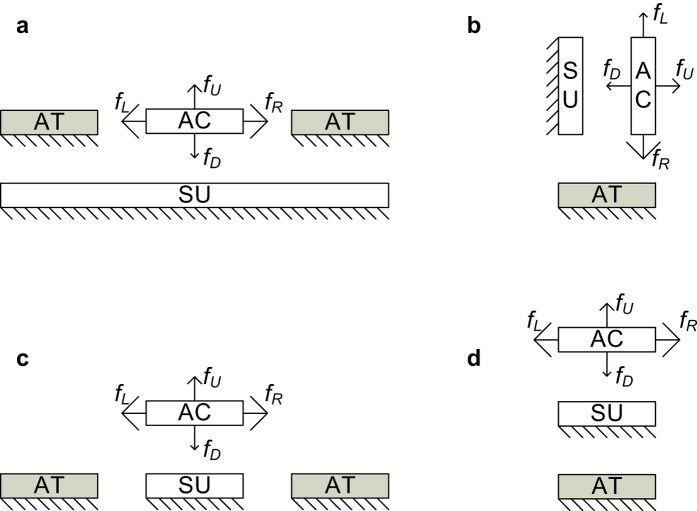
Various repulsive actuators. The solid rectangle AT is the attracting electrode, the fixed hollow rectangle SU is the suppressing electrode, and the third hollow rectangle AC is the actuated electrode. (**a**) Levitation of comb-drive actuators. (**b**) In-plane repulsive actuator proposed by Lee *et al*.^20^. The movement direction of AC is restricted to either left or right. (**c**) Out-of-plane repulsive actuator proposed by He *et al*.^21^. (**d**) Three-layered repulsive actuator proposed by Rezadad *et al*.^23^.

Figure 1d shows the basic structure of the three-layer repulsive actuator, which consists of three parallel electrodes with the same or similar width, length, and thickness. When appropriate voltages are applied, the movable upper electrode is driven to move vertically upward.

**Figure 2:**
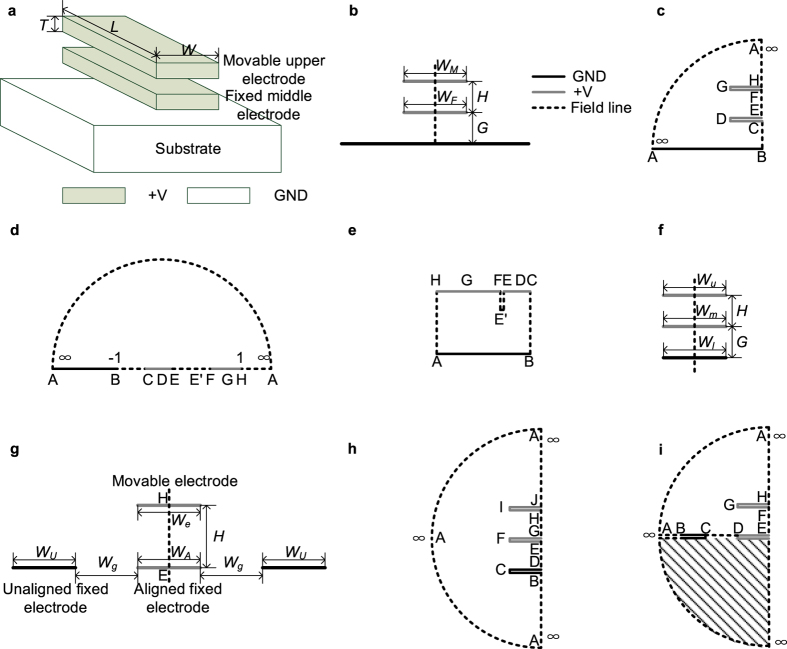
Structure and model of the three repulsive actuators. (**a**) 3D lateral view of the pseudo-three-layer actuator. (**b**) Cross-sectional view of (**a)**. (**c**) Left half of (**a)**. (**d**) Inverse SC mapping of (**c)**. (**e**) Forward SC mapping of (**d)**. (**f**) Cross-sectional view of the three-layer actuator. (**g**) Cross-sectional view of the two-layer actuator. (**h**) The model of (**f)**. (**i**) The model of (**g)**.

Although the three-layer actuator has a large fill factor, it requires three structural layers, which increase the difficulty of device fabrication. In most surface micromachining processes, the substrate is a doped semiconductor. Thus, it can be connected to the ground to serve as the lower electrode. The middle and upper electrodes can be deposited with two structural layers. This novel three-layer repulsive actuator, as shown in Fig. 2a, is called the pseudo-three-layer repulsive actuator in this paper.

The pseudo-three-layer repulsive actuator has the advantages of both the two-layer and the three-layer repulsive actuators. Its fabrication process is the same as that of the two-layer actuator, whereas its fill factor is the same as that of the three-layer actuator. In addition to these advantages, the most important characteristics of MEMS actuators are the force generated and their stroke. We therefore compare these three actuators, which are all exactly the same size, i.e., the widths of their electrodes and the gaps between them are identical, with regard to the driving force and the stroke.

### Modeling of the pseudo-three-layer actuator

First a theoretical model of the pseudo-three-layer actuator is constructed. In general, the electrode thickness is considerably small compared to the electrode length and width. Therefore, the thicknesses of all electrodes are neglected. Furthermore, the substrate is a conductor and its upper surface is an equipotential surface. When compared with the middle and upper electrodes, the length and width of the substrate can also be regarded as infinite. Given these two reasons, the electric field is limited above the upper surface of the substrate. Figure 2b shows the cross section of the pseudo-three-layer actuator, where the solid lines represent the equipotential surfaces and the dashed lines represent the electric field lines. The gap between the substrate and the middle electrodes, *G*, and the gap between the middle and upper electrodes, *H*, are two of the important actuator parameters. The value of *G* is fixed, and the value of *H* varies when the actuator is driven. Given the symmetry, the dashed line in Fig. 2b is the axis of symmetry.

The relationship between the electrostatic force and the variable *H* is derived. A technique that is similar to the method proposed by He *et al*. is used^26^. The electrostatic force is written as


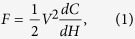


where *V* is the electric potential difference between AT and AC, and *C* is the capacitance between AT and the other two electrodes. In a practical actuator, *L* is considerably larger than *W*, *G*, and *H*. Therefore, the capacitance *C* can be obtained by multiplying electrode length *L* by the capacitance per unit length *C*_unit_:


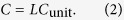


*C*_unit_ is the capacitance of the cross section shown in Fig. 2b. The field line in Fig. 2b divides the structure into two symmetrical parts, and each part has an identical capacitance *C*_half_. Therefore,


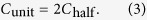


The dimensions of the structure are normalized with the width of the fixed middle electrode *W*_*F*_:


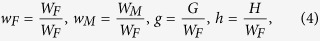


where *w*_*F*_, *w*_*M*_, *g*, and *h* are the normalized dimensions of *W*_*F*_, *W*_*M*_, *G*, and *H*. With expressions (1), (2), (3), and (4), the electrostatic force can be expressed as


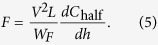


The computation of *C*_half_ is a 2D electrostatic field problem and is suitable for solution by conformal mapping. Figure 2c shows the left half of the pseudo-three-layer actuator. The closed curve composed of dashed lines and solid lines represents the region where the solution is sought. The middle and upper electrodes have no thicknesses. However, their upper and lower surfaces are drawn separately to indicate that the electrodes are excluded from the region in question.

Figure 2c illustrates a generalized polygon, and its vertices are denoted by A, B, …, H. A complex plane, Z-plane, is constructed such that vertex B is the origin and the vertical dashed line is the imaginary axis. Thus, the coordinates of all vertices can be derived in Z-plane based on the structural parameters. Through an inverse Schwarz–Christoffel (SC) transformation, Fig. 2c can be mapped to the upper half-plane of another complex plane, namely, W-plane, as shown in Fig. 2d. The coordinates of any three vertices in W-plane can be chosen arbitrarily, e.g., −1 (vertex B), 1 (vertex H), and infinity (vertex A). The following is the transformation function from W-plane to Z-plane^27^:


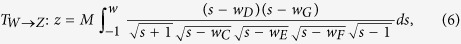


where *w*_*C*_, *w*_*D*_, *w*_*E*_, *w*_*F*_, and *w*_*G*_ are the complex coordinates of the corresponding vertices in W-plane. *M* is a complex constant. The complex coordinates of points C, D, E, F, G, H, and A in Z-plane, namely, *z*_*C*_, *z*_*D*_, *z*_*E*_, *z*_*F*_, *z*_*G*_, *z*_*H*_, and *z*_*A*_, respectively, are known, which are also the definite integrals of function (6) when their upper limits are *w*_*C*_, *w*_*D*_, *w*_*E*_, *w*_*F*_, *w*_*G*_, *w*_*H*_, and *w*_*A*_, respectively. These upper limits and *M* are then determined based on these relationships. No analytical solution exists for these coordinates. Therefore, in this paper, the complex coordinates of all vertices in W-plane are solved with an SC toolbox for MATLAB created by T. A. Driscoll^28^,^29^.

From vertex E to vertex F along the dashed line EF in Fig. 2c, the voltage first decreases and then increases. Therefore, a point E’ exists at which the minimum voltage occurs in line EF. Based on a forward SC transformation, the upper half-plane of W-plane can then be transformed into a folded rectangle in another complex plane, namely, Q-plane, as shown in Fig. 2e 30. The dashed lines EE’ and E’F overlap, although they are drawn separately to show that the structure is closed. This rectangle is a perfect (fringe effect-free) parallel-plate capacitor. The transformation function is


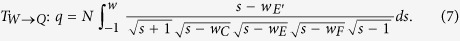


*w*_*E’*_ must be solved before the capacitance is computed. The fixed middle electrode, CE, has the same voltage as the movable upper electrode, FH. Thus, the length of EE’ should be equal to the length of E’F, and an equation can be written as


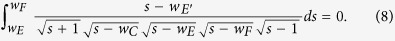


So *w*_*E*’_ can be expressed as


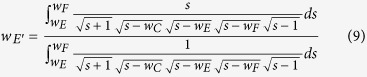


After solving for *w*_*E*’_, *q*_*A*_, *q*_*B*_, *q*_*C*_, and *q*_*H*_ can be calculated easily with function (7). Finally, given the invariance of the capacitance under a conformal transformation, the capacitance *C*_half_ can be expressed as


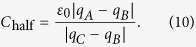


The complex constant *N* obviously cancels out. To compute the electrostatic force, the derivative in Equation (5) is approximated as


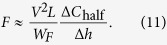


The interval of *h* is selected to be as small as 0.001. Then, electrostatic force *F* can be calculated based on Equations (10) and (11).

### Comparison with three-layer and two-layer actuators

Figure 2f shows the cross section of the three-layer actuator, which is modeled in Fig. 2h. The electrostatic force can also be calculated with the same method.

For the two-layer actuator, the definitions of all electrodes and sizes are shown in Fig. 2g. The dashed line in Fig. 2g is the axis of symmetry. The electric field line from the midpoint of the upper surface of the movable electrode (point H in Fig. 2g) to the grounded unaligned fixed electrode goes through an infinite distance. The field line from the midpoint of the lower surface of the aligned fixed electrode (point E in Fig. 2g) to the grounded unaligned fixed electrode also goes through an infinite distance. Therefore, the end of the field line reaching the grounded unaligned fixed electrode can be approximated symmetrically. The structure can then be modeled, as shown in Fig. 2i. According to the theoretical analysis of He *et al*.^26^, the field line between points C and D can be approximated as a straight line, as shown in Fig. 2i. In addition, the capacitance of the shaded region can be ignored. The final model is the generalized polygon ABCDEFGH.

**Figure 3:**
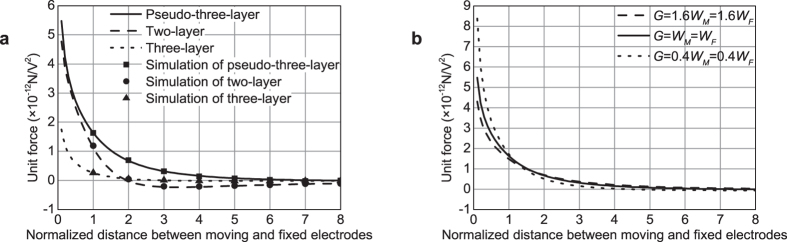
Analytical and simulation electrostatic force of the actuators. (**a**) Comparison of the three actuators. (**b**) Effect of *G* on the performance of the pseudo-three-layer actuator.

Based on the above analysis, the electrostatic forces of the three actuators which have exactly the same sizes, i.e., *G* = *W*_*u*_ = *W*_*m*_ = *W*_*l*_ = *W*_*M*_ = *W*_*F*_ = *W*_*U*_ = *W*_*A*_ = *W*_*e*_ = *W*_*g*_ = 1, are calculated and plotted in Fig. 3a. A numerical simulation is performed with the commercial software Maxwell SV^31^ to verify the theoretical results. All electrode thicknesses are set as 1% of its width to satisfy the presumption that electrode thicknesses are negligible. The simulation results are also shown in Fig. 3a.

As Fig. 3a shows, the pseudo-three-layer actuator has the highest electrostatic force among the normalized *h* ranges from 0.1 to 8. Its electrostatic force decreases to 0 until *h* is approximately 7.6. By contrast, the electrostatic forces of the two-layer and three-layer actuators reach 0 at *h* values of approximately 2 and 3.1 respectively. With the term of theoretical stroke introduced in ref. 26, the theoretical strokes for the pseudo-three-layer, two-layer, and three-layer actuators are obtained at 7.6, 2, and 3.1, respectively. Therefore, the pseudo-three-layer actuator has advantages not only in terms of fabrication requirements and fill factor, but also in terms of electrostatic force and theoretical stroke.

The preceding conclusion is only suitable for the case in which all the structural parameters of the three actuators are the same. However, in real devices, parameter *G* of the pseudo-three-layer and three-layer actuators is different from other parameters. It is mainly determined through fabrication process and cannot be tuned in design. In most surface micromachining processes, *G* is considerably smaller than the other parameters, which limits the vertical displacement range of the actuators. For the two-layer actuator, all structural parameters are determined with design and can be increased to achieve a large displacement. Therefore, on the basis of the preceding analysis, we also calculate the force in pseudo-three-layer actuator with different *G* values, as shown in Fig. 3b. The results show that as *G* decreases, the electrostatic force increases, while the position where the repulsive force decreases to 0 decreases. Further calculations indicate that when *G* = 0.03 *W*_*M*_ = 0.03 *W*_*F*_, the repulsive force reaches 0 at *h* value of approximately 2, which equals the turning point of *h* for the two-layer actuator. On the basis of these results, we can compare quantitatively the theoretical stroke of the pseudo-three-layer actuator with that of the two-layer actuator. For instance, in MUMPs process, where *G* equals 600 nm, when the width of all electrodes is smaller than 20 μm, the theoretical stroke of the pseudo-three-layer actuator is larger than that of the two-layer actuator. On the contrary, when the width is larger than 20 μm, the theoretical stroke of the two-layer actuator is greater than that of the pseudo-three-layer actuator.

**Figure 4:**
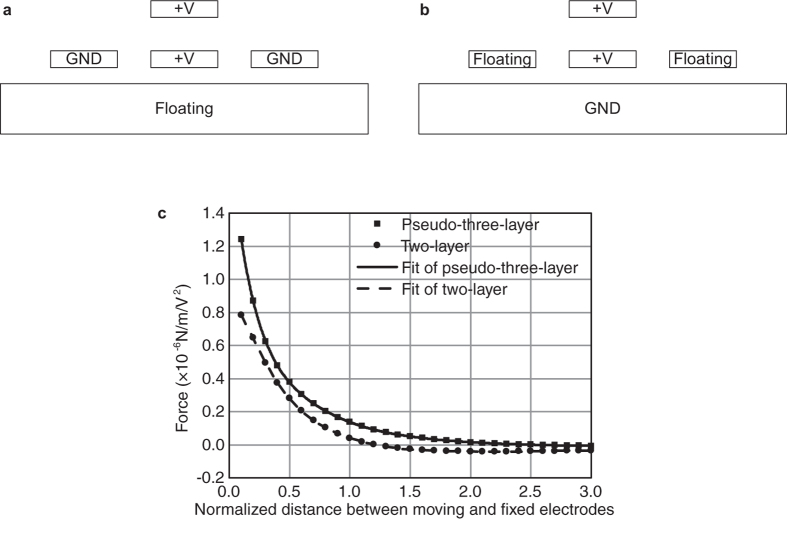
Two driving modes of the two-layer actuator. (**a**) Two-layer actuator connected in the two-layer driving mode. (**b**) Two-layer actuator connected in the pseudo-three-layer driving mode. (**c**) Comparison of electrostatic forces of the two connection modes.

In summary, the pseudo-three-layer repulsive actuator proposed in this paper has three main advantages:

1. It combines both advantages of three-layer actuator and two-layer actuator. In other words, it has a high fill factor similar to that of the three-layer actuator, and a low fabrication demand similar to that of the two-layer actuator.
2. It has the highest electrostatic force and theoretical stroke of the three actuators when they are of the same size.
3. Its structure is compatible with both the parallel-plate actuators and the two-layer and three-layer repulsive actuators. If the substrate meets the conductivity demand, these several types of actuators can then be connected to form pseudo-three-layer repulsive actuators. Take two-layer actuator as an example, the movable and aligned fixed electrodes and the substrate are treated as the three electrodes, and the unaligned fixed electrodes are left floating. Figure 4a,b show the two driving modes of the two-layer actuator. A device with this structure and its theoretical and experimental analysis is discussed in the next subsection.

### 19-element DM based on pseudo-three-layer repulsive actuator

**Figure 5:**
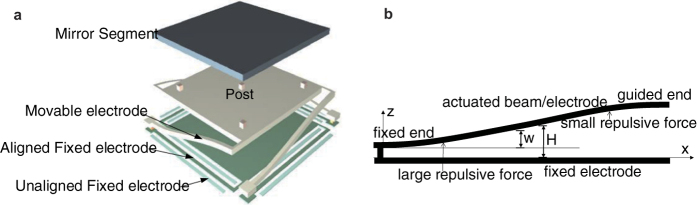
Structure and model of the DM. (**a**) Schematic diagram of DM. (**b**) The modeled flexural beam (movable electrode).

Our previously reported 19-element segmented DM^32^,^33^ based on two-layer repulsive actuator is utilized to verify the discussions in previous subsection. The DM was fabricated by MUMPs. One of the elements is shown in Fig. 5a.

The mirror segment is supported by four fixed-guided beams. At the same time, the beam forms the movable electrode of a two-layer repulsive actuator. One end of the beam is anchored to the substrate, and the other end is connected to the mirror segment. When the two-layer actuator is driven, the guided end of the four beams is actuated upward, and the mirror segment is moved upward in a translational manner. The structural parameters of the DM are listed in Table 1.

In MUMPs, the surface of the substrate, which is an n-type silicon wafer with a resistivity of 1–2 Ω-cm, is doped with phosphorus oxychloride (POCl_3_) in the first step of the process. After POCl_3_ doping, the substrate surface resistance is 9–10 Ω/square, which can be regarded as conductive. Then, the four two-layer repulsive actuators of DM element can be connected as a pseudo-three-layer repulsive actuator, as shown in Fig. 4b.

When the actuator is connected as a two-layer structure or a pseudo-three-layer structure, the movable electrode is deformed in both cases as a fixed-guided beam, as shown in Fig. 5b. The distance between this electrode and the aligned fixed electrode is then varied along the longitudinal direction, which results in a change of the electrostatic force along the beam.

Given the advantages of repulsive actuators, the deflection is comparable with the thickness of the 2 μm movable electrode. Therefore, large deflection theory must be included here. As a result, the deflection should satisfy^34^


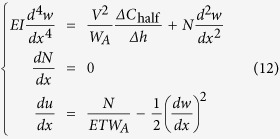


where *E*, *I*, *w*, and *u* represent the Young’s modulus, moment of inertia, deflection in the *z*-direction of the movable electrode, and deflection in the *x*-direction of the movable electrode, respectively. *N* represents the axis force induced by the large deflection. Additionally, the following six boundary conditions exist:


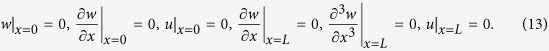


These boundary conditions represent 1) the fixed end of the beam having no deflection in the *z*-direction and no slope and no deflection in the *x*-direction, and 2) the guided end of the beam having no slope, no concentrated force, and no deflection in the *x*-direction.

The first term on the right side of the first equation of Equation (12), i.e., the electrostatic force 1/*W*_*A*_·Δ*C*_half_/Δ*h*, can be solved using the proposed model. The values of the two driving modes are calculated based on the structural parameters and are plotted in Fig. 4c.

To express the first equation of Equation (12) analytically, the least squares method is used to fit the calculated values with a polynomial expression. The polynomial approximation is


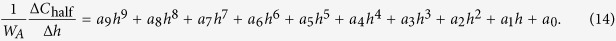


The coefficients *a*_0_, …, *a*_9_ have units of N/m/V^2^ and are listed in Table 2.

The relationship between the deflection in the *z*-direction, *w*, and the normalized height, *h*, is given as


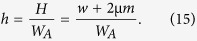


**Figure 6:**
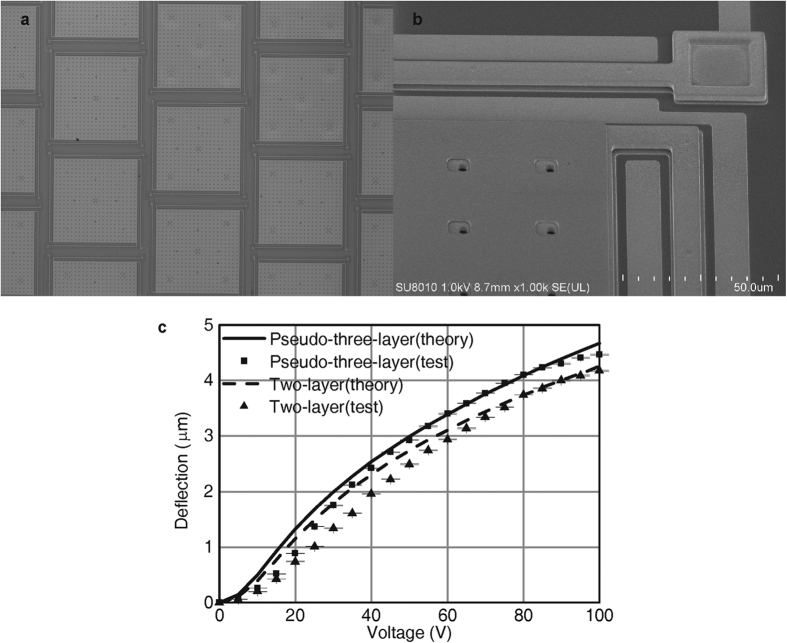
Theoretical and experimental results of the DM. (**a**) Micrograph of the DM. (**b**) SEM photograph of the DM. (**c**) Comparison of displacements of the two connection modes.

The relationship between *w* and *V* can be determined by substituting expressions (14) and (15) into (12) and by solving Equations (12) and (13) simultaneously with MATLAB. These theoretical results are drawn in Fig. 6c.

Figure 6a,b show a micrograph and the scanning electron microscopy (SEM) images of the repulsive DM. The four electrodes can be clearly seen in Fig. 6b.

The voltage-deflection performance of the DM is measured with a 3D optical profiler, namely, Zygo NewView 7300 (Zygo, Middlefield, CT, USA). First, one of the elements is connected in the two-layer mode and dc voltages of 0–100 V are applied with increments of 5 V. The measured deflections are shown in Fig. 6c (The values are averaged over 5 independent measurements). Then, the same element is connected in the pseudo-three-layer mode, and the same voltages are applied again. The new voltage-deflection relationship is also shown in Fig. 6c.

From the experimental results, it is easy to see that in the pseudo-three-layer mode, the deflection is larger, and a maximum deflection of 4.5 μm is achieved. Several other elements are also measured and, deflections in the range of 4.1–4.5 μm are achieved under a driving voltage of 100 V.

Overall, the theoretical results are consistent with the experimental results. The discrepancies are mainly attributed to two reasons: 1) The potential of the mirror segment is the same as that of the movable electrode, which alters the electric field distribution and slightly reduces the repulsive force. 2) The initial bending deformation of the large mirror segment exists because of liquid surface tension effects when the structure is released in the HF solution, thereby modifying the initial height of the movable electrode.

## Discussions

In summary, a pseudo-three-layer repulsive actuator that integrates the advantages of the two-layer and the three-layer repulsive actuators is proposed. A theoretical analysis that combines physical modeling with SC numerical calculations is performed. The pseudo-three-layer actuator has the largest electrostatic force and theoretical stroke when compared with the other two actuators. Its structure is compatible with the parallel-plate actuators and the repulsive actuators, and existing devices based on these actuators can be altered to easily accommodate the pseudo-three-layer actuator. A previous 19-element DM based on a two-layer actuator is converted into the pseudo-three-layer mode, and the resulting device is analyzed theoretically and experimentally. The results show a larger displacement than that in the two-layer mode under the same applied voltage and a maximum displacement of 4.5 μm is achieved.

## Methods

### Sample fabrication

The DM was fabricated by MUMPs, which has the general features of a standard surface micromachining process. Three polysilicon layers (Poly 0–2) are used as the structural material, two deposited oxide (PSG) layers are applied as the sacrificial material, and silicon nitride is used as electrical isolation between the polysilicon and the substrate, and a metal layer for electronic connection and optical reflection.

## Additional Information

**How to cite this article**: Wang, W. *et al*. Electrostatic repulsive out-of-plane actuator using conductive substrate. *Sci. Rep*. **6**, 35118; doi: 10.1038/srep35118 (2016).

## Figures and Tables

**Table 1:** Physical parameters of 19-element DM.

Symbol	Quantity	Value
*W*	width of mirror segment	600 μm
*L*	length of movable electrode	620 μm
*W*_*e*_	width of movable electrode	9 μm
*W*_*U*_	width of unaligned fixed electrode	11 μm
*W*_*A*_	width of aligned fixed electrode	11 μm
*T*	thickness of movable electrode	2 μm
*H*_*init*_	initial height between movable and aligned fixed electrodes	2 μm
*G*	gap between the fixed electrodes and substrate	600 nm
*W*_*g*_	gap between the adjacent fixed electrodes	3 μm
*E*	Young’s modulus of the beam	160 GPa

**Table 2:** Coefficients of the polynomial approximation.

Fitting coefficient	Two-layer	Pseudo-three-layer
*a*_0_	8.90567 × 10^−7^	1.84994 × 10^−6^
*a*_1_	−6.78111 × 10^−7^	−7.60308 × 10^−6^
*a*_2_	−5.24223 × 10^−6^	1.77574 × 10^−5^
*a*_3_	1.54741 × 10^−5^	−2.60831 × 10^−5^
*a*_4_	−2.07575 × 10^−5^	2.46198 × 10^−5^
*a*_5_	1.6179 × 10^−5^	−1.51109×10^−5^
*a*_6_	−7.70927 × 10^−6^	5.9924 × 10^−6^
*a*_7_	2.21178 × 10^−6^	−1.48008 × 10^−6^
*a*_8_	−3.50891 × 10^−7^	2.06956 × 10^−7^
*a*_9_	2.3647 × 10^−8^	−1.25103 × 10^−8^
